# Colorectal cancer and *Cryptosporidium* spp. infection

**DOI:** 10.1371/journal.pone.0195834

**Published:** 2018-04-19

**Authors:** Violetta Sulżyc-Bielicka, Lidia Kołodziejczyk, Sylwia Jaczewska, Dariusz Bielicki, Krzysztof Safranow, Paweł Bielicki, Józef Kładny, Wojciech Rogowski

**Affiliations:** 1 Department of Clinical Oncology, Pomeranian Medical University, Szczecin, Poland; 2 Chair and Department of Biology and Medical Parasitology, Pomeranian Medical University, Szczecin, Poland; 3 Department of Clinical Oncology, Public Hospital, Szczecin, Poland; 4 Department of Gastroenterology, Pomeranian Medical University, Szczecin, Poland; 5 Department of Biochemistry and Medical Chemistry, Pomeranian Medical University, Szczecin, Poland; 6 West Pomeranian Oncological Center, Szczecin, Poland; 7 Department of General and Oncological Surgery, Pomeranian Medical University, Szczecin, Poland; 8 Department of Clinical Oncology, Magodent Warszawa, Poland; University of Nebraska Medical Center, UNITED STATES

## Abstract

Transient or constant impaired immunity is often associated with neoplastic disease or oncological treatment. Among the most common pathogens found in patients with HIV or patients undergoing chemotherapy are protozoans of the *Cryptosporidium* genus, which cause diarrhea in humans and animals. The present study determined the frequency of *Cryptosporidium* spp. infections in patients with colorectal cancer (N = 108; 42 women; 66 men; median age, 65 years), before beginning oncological treatment, compared to a control group (N = 125; 56 women, 69 men; median age, 63 years) without colorectal cancer or a history of oncological disease. We also assessed whether *Cryptosporidium* spp. infections were associated with age, gender, cancer stage (based on Astler-Coller or TNM classification), histological grade, or cancer location. Patients were treated at the Pomeranian Medical University, in 2009–2014. The presence of *Cryptosporidium* spp. antigen was determined in stool samples, analyzed with a commercial immunoenzymatic test. *Cryptosporidium* spp. infections occurred significantly more often (p = 0.015) in patients (13%) compared to controls (4%). The patient group showed no significant relationship between *Cryptosporidium* spp. infection and sex, age, tumor location, cancer grade, or stage. A multivariate logistic regression analysis adjusted for age and sex that included all subjects (patient + control groups, n = 233) showed that the odds of a *Cryptosporidium* spp. infection were more than three-fold higher in patients than in controls, and more than six-fold higher among men than among women. Conclusions: 1) *Cryptosporidium* spp. infections occurred significantly more frequently in patients with colorectal cancer (before oncological treatment) compared to controls, independent of age and sex. 2) *Cryptosporidium* spp. infections were not associated with the colorectal cancer stage, grade, or location or with patient age. 3) Male gender was significantly related to the frequency of *Cryptosporidium* spp. infections, independent of age and the presence of colorectal cancer.

## Introduction

Colorectal cancer is one of the most common malignant neoplasms in humans. Its incidence is the third highest among malignant neoplasms worldwide, and it accounts for 9.7% of all tumors [1]. Colorectal cancer arises from an accumulation of genetic and epigenetic changes that transform healthy epithelial cells into cancer cells. In addition to genetic factors, environmental factors contribute to the genesis of colorectal cancer, like red meat-rich diets, low physical activity, and chronic nicotine use [2]. There is also evidence that bacteria in intestinal flora play a significant role in colorectal cancer development [3].

*Cryptosporidium* is a small protozoan that infects cells and exists intracellularly, particularly in the gastrointestinal tract epithelium of vertebrates, including birds, reptiles, amphibians, humans, and other mammals. The *Cryptosporidium* infection is one of the most important etiologies of diarrhea among humans [4]. It was estimated that *Cryptosporidium* infections accounted for 1-3% of diarrhea incidences in the US and Europe and 10–15% of incidences in developing countries [5]. Most infections in humans are caused by *C*. *parvum* and *C*. *hominis* [6]. These are responsible for over 90% of all cases [6]. Invasive forms (oocysts) are resistant to external factors and water treatment processes. They can survive in temperatures of -20°C and in salt water [7]. The infection spreads in a fecal-oral pathway. It is mostly transmitted through contaminated food or water, but it can also be transmitted through direct contact with infected humans or animals, and rarely, through inhalation. Cryptosporidiosis can occur sporadically or in an epidemic. In the last several years, more than 100 epidemics have been detected and described [8]. Among these, a Cryptosporidiosis outbreak occurred in Milwaukee, which affected 403 000 people [9]. *Cryptosporidium* might also be responsible for opportunistic infections among patients with neoplasms [10, 11].

Among patients with immunocompetence, exposure to *Cryptosporidium* results in an infection of the small intestine that may be asymptomatic or it may cause transient, watery-mucous diarrhea, which resolves without treatment. The diarrhea might be accompanied by nausea, vomiting, abdominal pain, and sometimes fever. The infection causes transient destruction of microvilli and crypt hyperplasia with inflammatory cell infiltration. Patients with a competent immune system quickly recover from cryptosporidiosis, typically within two weeks. However, in patients with damaged, underdeveloped (e.g., infants and small children), or compromised immune systems (e.g., patients with AIDS), infections can lead to chronic diarrhea, water-electrolyte imbalances, malnutrition, and even death [12,13].

According to International Agency for Research on Cancer (IARC), there are 11 biological factors that are carcinogenic in humans. These factors are: EBV, HBV, HCV, HHV-8, HIV, HPV, HTLV-1, *Helicobacter pylori*, *Schistosoma haematobium*, *Opisthorchis viverrini*, and *Clonorchis siniensis* [14]. Some of anti-infective therapies may lead to the remission of malignancy as in case of gastric MALT lymphoma. Current guidelines indicate *H*. *pylori* eradication therapy as the first line approach in gastric MALT lymphoma [15,16].

The role of protozoa in tumor growth remains unknown. IARC has not considered protozoans carcinogenic for humans. However, the available data show that some protozoa may be associated with tumor growth, including: *Trichomonas vaginalis*, *Toxoplasma gondii*, and *C*. *parvum* [17]. The notion that a chronic *Cryptosporidium* infection might influence tumor development is based on limited data, and those data were mainly from studies conducted on animals [18, 19, 20, 21].

Cryptosporidiosis, an opportunistic infection, is best-known and documented in patients with HIV infections. Patel et al. found significantly more occurrences of colorectal cancer among patients with HIV infections than in the general population [22]. Shebl et al. [23] showed that the risk of colorectal cancer was higher among patients with HIV infections and cryptosporidiosis than among patients with HIV infections without cryptosporidiosis. That finding indirectly suggested that *Cryptosporidium* infections might be associated with the development of colorectal cancer. Moreover, statistically significant differences were found among patients with planoepithelial cancer and patients with other rare types of colorectal cancer [23].

The present study had two main objectives. First, we determined the frequency of *Cryptosporidium* spp. infections in patients with colorectal cancer, before beginning oncological treatment. The results were compared to results in a control group of individuals without cancer after adjustment for age and gender. Second, we assessed whether the occurrence of *Cryptosporidium* spp. infections was associated with the age or gender of patients, the colorectal cancer stage, the histological grade, or the location of cancer.

## Materials and methods

This study included 108 consecutive patients with colorectal cancer that were hospitalized from 2009–2014 in the Department of General and Oncological Surgery, Pomeranian Medical University. The group had a median age of 65 years and consisted of 42 women (38.9%) and 66 men (61.1%). The colonoscopy examination was conducted in the Department of Gastroenterology Pomeranian Medical University. All patients were diagnosed based on histopathological confirmations of cancer. Patients with concomitant neoplasms or with a history of another cancer were excluded from the study. No patient included in the study had previously undergone chemotherapy. Details of the patient characteristics are shown in Table 1.

**Table 1:** Characteristics of patients with colorectal cancer (n = 108).

Examined parameters	Values
Age (years); Median (range)	65 (38–88)
Gender:	
female	42 (38.9)
male	66 (61.1)
Tumor location:	
right side of colorectum^a^	25 (23.1)
left side of colorectum^b^	83 (76.9)
Tumor location:	
rectum	45 (41.7)
colon	63 (58.3)
TNM Staging:	
I	18 (16.7)
IIA	36 (33.3)
IIB	0
IIC	1 (0.9)
IIIA	1 (0.9)
IIIB	29 (26.9)
IIIC	7 (6.5)
IV	16 (14.8)
Astler Coller staging:	
A	3 (2.8)
B1	15 (13.9)
B2	36 (33.3)
B3	1(0.9)
C1	2 (1.8)
C2	33 (30.6)
C3	2 (1.9)
D	16 (14.8)
Astler Coller staging:	
A+B	55 (50.9)
C+D	53 (49.1)
Grading*:	
G1	7 (6.6)
G2	78 (73.6)
G3	14 (13.2)
Mucinosum histology	7 (6.6)
Grading*:	
G1+G2	85 (80.2)
G3+mucinosum histology	21 (19.8)

Values indicate the number of patients (%), unless indicated otherwise.

^a^Right-side location, including the cecum, colon ascendens, and colon transversum

^b^Left-side location, including the colon descendens, sigmoid, sigmoid-rectal flexure, and rectum; TNM: tumor size, nodal involvement, and metastasis grading

*G-grading was absent in two patients

We also recruited a control group of 125 individuals without a diagnosis of colorectal cancer and no history of cancer at the time of interview. These included 56 women (44.8%) and 69 men (55.2%). The median age was 63 years.

On the day of admission to the Department of General and Oncological Surgery, stool samples were collected from patients with colorectal cancer. Stool samples were also collected from the control group in the Department of Gastroenterology, Pomeranian Medical University. All samples were delivered to the Department of Biology and Medical Parasitology, Pomeranian Medical University. The study was approved by the Bioethics Committee Pomeranian Medical University (KB-0012/179/05/16). According to Bioethics Committee instructions, verbal consents were obtained from patients because of non-invasive character of the conducted study. Examinations were made additionally to routine stool-sample investigation for parasite infection. Attestation statements of verbal consents of all participants in conducted study, signed by the physicians were gathered in medical record.

### Immunoenzymatic test for *Cryptosporidium* (Antigen-EIA technique)

Stool samples were analyzed to determine the presence or absence of *Cryptosporidium* spp. antigen, with a commercial immunoenzymatic test (ProSpecT Cryptosporidium Microplane Assay), according to the manufacturer’s instructions. The test detected CSA, an antigen specific to *Cryptosporidium*. The intensity of the reaction product was analyzed with a visual method.

### Statistical analysis

Associations between *Cryptosporidium* spp. infection and qualitative variables were evaluated with Pearson’s chi-squared test or Fisher’s exact test. Associations of *Cryptosporidium* spp. infection with age and rank variables (e.g., cancer stage) were analyzed with the Mann-Whitney U test. A multivariate logistic regression analysis was conducted to identify independent risk factors for *Cryptosporidium* spp. infections. The threshold for statistical significance was p<0.05. The calculations were performed with Statistica 10 (StatSoft Inc., USA) and Microsoft Excel 2003.

## Results

We found no significant differences in age and gender between the colorectal cancer (n = 108) and control (n = 125) groups (Table 2). *Cryptosporidium* spp. infections occurred significantly more frequently in the colorectal cancer group than in the control group (p = 0.015, Table 2). The presence of *Cryptosporidium* spp. antigen was detected in 14 (13%) patients with colorectal cancer and in 5 (4%) individuals in the control group.

**Table 2:** Comparison of the characteristics and frequencies of *Cryptosporidium* spp. infections in colorectal cancer (CRC; n = 108) and control (n = 125) groups.

Parameter	CRC group	Control group	p
Age (years); Median (range)	65 (38–88)	63 (38–88)	0.175^a^
Gender:			
female	42 (38.9)	56 (44.8)	0.424^b^
male	66 (61.1)	69 (55.2)	
*Cryptosporidium* spp. infection:			
current infection	14 (13)	5 (4)	0.015^b^
current lack of *Cryptosporidium* infection	94 (87)	120 (96)	

Values indicate number of patients (%), unless indicated otherwise.

^a^Mann-Whitney U test

^b^Fisher’s exact test

In the colorectal cancer group, we analyzed associations between *Cryptosporidium* spp. infection and age, sex, cancer stage (according to the TNM and Astler-Coller classifications), histological grade, and tumor location (in the rectum or colon; on the left-side or right-side of the splenic flexure in the colon). We found no significant associations between *Cryptosporidium* spp. infection and any of these factors (Table 3). However, according to the Astler-Coller classification, the median cancer stages were C2 (IIIB according to TNM classification) for individuals infected and B2 (IIA) for those not infected. Nevertheless, those results were not statistically significant (respectively: p = 0.642 and p = 0.674).

**Table 3:** Associations between *Cryptosporidium* spp. infection (CR) and various clinical parameters in the group of individuals with colorectal cancer (n = 108).

Parameter	No CR, N (%)	With CR, N (%)	p
Age (years); Median (range)	64 (40–88)	69 (38–81)	0.323^b^
Gender:			
female	40 (95.2)	2 (4.8)	0.075^a^
male	54 (81.8)	12 (18.2)	
Staging according to Astler-Coller:			
A	3 (100)	0	0.735^b^
B1	14 (93.3)	1 (6.7)	
B2	31 (86.1)	5 (13.9)	
B3	1 (100)	0	
C1	2 (100)	0	
C2	26 (78.8)	7 (21.2)	
C3	2 (100)	0	
D	15 (93.7)	1 (6.3)	
Staging according to Astler-Coller:			
A+B	49 (89.1)	6 (10.9)	0.576^a^
C+D	45 (84.9)	8 (15.1)	
Staging according to TNM:			
I	17 (94.4)	1 (5.6)	0.674^b^
IIA	31 (86.1)	5 (13.9)	
IIC	1 (100)	0	
IIIA	1 (100)	0	
IIIB	23 (79.3)	6 (20.7)	
IIIC	6 (85.7)	1 (14.3)	
IV	15 (93.7)	1 (6.3)	
Grading:			
G1	7 (100)	0	0.48^c^
G2	66 (84.6)	12 (15.4)	
G3	12 (85.7)	2 (14.3)	
Mucinosum histology	7 (100)	0	
Grading:			
G1+G2	73 (85.9)	12 (14.1)	0.731^a^
G3+mucinosum histology	19 (90.5)	2 (9.5)	
Tumor location:			
left-side	71 (85.5)	12 (14.5)	0.514^a^
right-side	23 (92)	2 (8)	
Tumor location:			
rectum	39 (86.7)	6 (13.3)	1.0^a^
colon	55 (87.3)	8 (12.7)	

Values indicate the number of patients (%), calculated per row, unless indicated otherwise. CR: *Cryptosporidium* spp. infection

^a^Fisher’s exact test

^b^Mann-Whitney U test

^c^chi-square test

A similar analysis of the control group (n = 125) showed no association between *Cryptosporidium* spp. infection and age or gender. However, in this group, *Cryptosporidium* spp. infections were detected in 5 men and no women. Nevertheless, this difference did not reach statistical significance threshold (Table 4).

**Table 4:** Associations between *Cryptosporidium* spp. infection (CR) and age and gender, in the control group (n = 125).

Parameter	No CR	With CR	p
Age (years); Median (range)	63 (38–88)	62 (52–74)	0.781^b^
Gender, number (%):			
female	56 (100)	0	0.064^a^
male	64 (92.7)	5 (7.3)	

Values indicate the number of patients (%), calculated per row, unless indicated otherwise; CR: *Cryptosporidium* spp. infection

^a^Fisher’s exact test

^b^Mann-Whitney U test

In a multivariate logistic regression analysis, we analyzed the age and gender of all individuals in the experimental and control groups (n = 108+125). We found that the odds of *Cryptosporidium* spp. infection were over 3-fold higher in patients with colorectal cancer than in control subjects (Table 5). The model showed that male gender was a significant independent risk factor for *Cryptosporidium* spp. infection. The odds of infection among men were more than 6-fold higher than among women. We detected no relationship between *Cryptosporidium* spp. infection and participant age (Table 5).

**Table 5:** Multivariate logistic regression results of associations between *Cryptosporidium* spp. infection and gender, age, and colorectal cancer diagnosis (both groups, n = 233).

Independent variables	Odds Ratio (95% Confidence Interval)	p
Age	1.003 (0.956–1.053)	0.883
Gender: men vs. women	6.631 (1.468–29.954)	0.013
Colorectal cancer: patient group vs. control group	3.428 (1.167–10.067)	0.024

## Discussion

Patients with tumors often experience transient or constant impairments in immunity, caused by either the neoplastic disease or the oncological treatment. Among the most common pathogens in patients with HIV or those undergoing chemotherapy are the protozoans of the *Cryptosporidium* genus [11, 24, 25]. The immune response associated with cryptosporidiosis involves cellular and humoral components. The T-lymphocyte cellular responses are crucial in controlling infection, as evidenced by the increased disease severity in HIV-infected patients with CD4 counts less than 100 cells/microL [26, 27]. Specific IgM, IgG, and/or IgA responses develop during infection. The study of young children infected with *Cryptosporidium* in Haiti demonstrated the increased presence of systemic and intestinal proinflammatory cytokines (eg, tumor necrosis factor and interleukin-8) compared to healthy controls [28]. On the other hand the production of IFN-gamma is involved in the resolution of infection [29, 30].

The present study showed that *Cryptosporidium* spp. infections occurred significantly more often in patients with colorectal cancer, at the time of diagnosis, than in a control group without a neoplastic disease. The relatively high proportion of *Cryptosporidium* spp. infections (13%) found among patients with colorectal cancer at the time of diagnosis was consistent with findings in our earlier pilot reports (18% and 12.6%) [31, 32]. The present study showed that the odds of contracting a *Cryptosporidium* spp. infection were 3.4-fold higher in patients with colorectal cancer, before treatment, than among individuals without a neoplasm. To our knowledge, this study was the first in Europe which evaluated *Cryptosporidium* spp. infections prevalence in group of individuals with colorectal cancer before oncological treatment.

Most previous studies were conducted with patients during chemotherapy, which leads to conditions that foster the development of opportunistic infections [11, 24, 25]. Among those studies, the prevalence of *Cryptosporidium* infections varied from 1.3% to 80% [11, 24, 25, 33, 34, 35, 36]. The wide discrepancies in prevalence could be due to differences in the selected groups of patients, the regions the studies were conducted, or the diagnostic methods used to detect *Cryptosporidium*. Of note, in the majority of studies, cryptosporidiosis was described as a consequence of derivative immune deficiency, caused by the tumor and the treatment. Therefore, the question remained for patients with colorectal cancer: was the *Cryptosporidium* infection associated with the immune disorder, as is commonly found among patients with cancer? Or was there a relationship between the *Cryptosporidium* infection and colorectal cancer development?

A recent literature review showed that studies that investigated the influence of chronic *Cryptosporidium* infections on colorectal cancer growth were conducted mainly in animals. In 2007, Certad et al. showed that, in mice with severe combined immunodeficiency (SCID), an infection of *C*. *parvum* was associated with the growth of adenoma and adenocarcinoma in the digestive tract [18]. Their study was repeated in 2010, and additionally, they checked the Ki-67 proliferation marker. That study demonstrated a significant relationship between the severity of Cryptosporidiosis and the malignancy of tumors [19, 20]. In another study, Benamrouz et al. showed that, in mice with SCID, an infection with one oocyst of *C*. *parvum* led to the growth of adenocarcinoma in the digestive tract [37]. Moreover, Certad et al. also found an association between *C*. *parvum* infections and neoplastic growth among mice with SCID treated with dexamethazone [19, 38]. In mice infected with *C*. *parvum*, adenomas with intraepithelial and intramucosal neoplasias of different grades have been detected in the digestive tract, including the duodenum, stomach, and caecum [18, 19, 20]. Abdu et al. [39] assessed the relationship between dysplastic changes that developed in the intestine and *Cryptosporidium* infections in mice. They demonstrated that dysplastic changes only developed in infected mice, independent of an immune deficiency. The dysplastic changes were not detected in groups treated with nitazoxanide [39].

An interesting case report described an individual with lymphoblastic leukemia that developed severe Cryptosporidiosis after a bone marrow transplant [37]. Mice with SCID were infected with the *Cryptosporidium* strain isolated from this patient. Those mice rapidly developed severe cryptosporidiosis and generalized neoplasms in the stomach, intestine, and biliary tract. Neoplasms were detected in each location that protozoa were detected. Thus, the *Cryptosporidium* isolated from a human led to cancer growth in an experimental mouse model, which suggested that this protozoa might directly impact the carcinogenic process [37]. However, the pathomechanism underlying the potential impact of a *Cryptosporidium* infection on the genesis of colorectal cancer remains to be clarified. Experimentally, a chronic *Cryptosporidium* infection was shown to impact the expression of many genes in host cells, including genes involved in apoptosis [39, 40, 41]. Benamrouz et al. showed that mice infected with *C*. *parvum* exhibited impaired expression of genes that encoded APC and ß-catenin, two components of the Wnt pathway [21].

The present study showed that, among all subjects (patients with colorectal cancer and controls), male gender was an independent factor associated with a high prevalence of *Cryptosporidium* spp. infections. The odds of *Cryptosporidium* spp. infections were 6.6-fold higher among men than among women. The European registry indicated that the ratio of men to women with *Cryptosporidium* infections varied with the country, but it ranged between 0.8 and 1.5 (European Center for Disease Prevention and Control; report from 2014). Clinical and preclinical studies have indicated that there are sex- and gender-associated differences in colorectal cancer pathogenesis. Women have a higher risk of developing right-sided colon cancer than men, which is associated with worse clinical outcome compared to left-sided tumors. Both genetic and environmental factors are believed to play roles in sex and gender differences in right- *vs* left-sided colon cancers [42]. Sharma A et al. showed that pre-operative IL-1beta and post-operative IL-6 levels were significantly higher in males compared with females with colorectal cancer. This provides evidence of a possible link between gender and cytokine levels in patients with colorectal cancer [43].

Recent studies have shown differences of the immune system responds to disease and therapy. T helper1 (Th1) and Th2 cytokines (Th1/Th2) have pivotal roles in the homeostasis of Th1 and Th2 cell network functions in the immune response but sex steroids affect Th1/Th2 production in different ways and a natural sexual dimorphism of the immune response has been shown. The results show that the interferon γ production pathway for immune response homeostasis is specific to men whilst the IL-6 production pathway for immune response homeostasis is specific to women. The IL-10 pathway for restoring immune system resting homeostasis was common to both but was controlled by the respective gender-specific pathways [44].

Latest findings indicate that commensal microbial imbalance (dysbiosis) can trigger DNA damage response (DDR) activation in host cells, which may result in sustained inflammatory responses. Therefore, dysbiosis can be seen as an important source of DNA damage agents that may be partially responsible for the overexpression of NKG2D-Ls on intestinal epithelial cells that is frequently observed also in patients with disorders associated with altered human microbiota, including the development of colorectal cancer [45].

The findings of the present study led to the following conclusions:

1. *Cryptosporidium* spp. infections appeared significantly more often in patients with colorectal cancer, prior to oncological treatment, than in controls, independent of age and sex.
2. Among patients with colorectal cancer, the *Cryptosporidium* spp. infection was not associated with the colorectal cancer stage, grade, or location, or with patient age.
3. Male gender was significantly related to an increased frequency of *Cryptosporidium* spp. infections, independent of age and the presence of colorectal cancer.

Further investigation is required to determine the temporal and causal relationships between the development of colorectal cancer and *Cryptosporidium* spp. infections in humans.

## Supporting information

S1 Data Raw data analyzed in the current manuscript are available in attached “Sulzyc Bielicka database.xls” file. (XLS)

## References

[1] Ferlay J, Shin HR, Bray F, Forman D, Mathers C, Parkin DM. Estimates of worldwide burden of cancer in 2008: GLOBOCAN 2008. Int J Cancer. 2010;127: 2893–2917. doi: 10.1002/ijc.25516 21351269 10.1002/ijc.25516

[2] Kerr J, Anderson C, Lippman SM.: Physical activity, sedentary behaviour, diet, and cancer: an update and emerging new evidence. Lancet Oncol. 2017; 18: e457–e471. doi: 10.1016/S1470-2045(17)30411-4 28759385 10.1016/S1470-2045(17)30411-4 PMC10441558

[3] Keku TO, Dulal S, Deveaux A, Jovov B, Han X. The gastrointestinal microbiota and colorectal cancer. Am J Physiol Gastrointest Liver Physiol. 2015; 308: G351–363. doi: 10.1152/ajpgi.00360.2012 25540232 10.1152/ajpgi.00360.2012 PMC4346754

[4] Shrivastava AK, Kumar S, Smith WA, Sahu PS. Revisiting the global problem of cryptosporidiosis and recommendations. Trop Parasitol. 2017; 7: 8–17. doi: 10.4103/2229-5070.202290 28459010 10.4103/2229-5070.202290 PMC5369280

[5] Lima AAM, Samie A, Guerrant RL. Cryptosporidiosis In: Guerrant RL, Walker DH, Weller PF, editors. Tropical Infectious Diseases. Philadelphia: Elsevier-Churchill Livingstone, 2011, pp. 640–663.

[6] Xiao L. Molecular epidemiology of cryptosporidiosis: An update. Exp. Parasitol. 2010; 124, 80–89. doi: 10.1016/j.exppara.2009.03.018 19358845 10.1016/j.exppara.2009.03.018

[7] Carey CM, Lee H, Trevors JT. Biology, persistence and detection of *Cryptosporidium* parvum and Cryptosporidium hominis oocyst. Water Res. 2004; 38: 818–62. doi: 10.1016/j.watres.2003.10.012 14769405 10.1016/j.watres.2003.10.012

[8] Putignani L, Menichella D. Global distribution, public health and clinical impact of the protozoan pathogen *Cryptosporidium*. Interdiscip Perspect Infect Dis. 2010; 2010. pii: 753512. 10.1155/2010/753512 PMC2913630 20706669

[9] Mac Kenzie WR, Hoxie NJ, Proctor ME, Gradus MS, Blair KA, Peterson DE, et al A massive outbreak in Milwaukee of *Cryptosporidium* infection transmitted through the public water supply. N Engl J Med. 1994; 331: 161–167. doi: 10.1056/NEJM199407213310304 7818640 10.1056/NEJM199407213310304

[10] Desoubeaux G1, Caumont C, Passot C, Dartigeas C, Bailly E, Chandenier J, Duong TH. Two cases of opportunistic parasite infections in patients receiving alemtuzumab. J Clin Pathol. 2012; 65: 92–5. doi: 10.1136/jclinpath-2011-200403 22052936 10.1136/jclinpath-2011-200403

[11] Rivera WL, Yason JA, Rivera PT. Serological detection of cryptosporidiosis among Filipino cancer patients. Parasitol Res. 2005; 98: 75–6. doi: 10.1007/s00436-005-0014-x 16237576 10.1007/s00436-005-0014-x

[12] Widerström M, Schönning C, Lilja M, Lebbad M, Ljung T, Allestam G, et al Large Outbreak of *Cryptosporidium hominis* Infection Transmitted through the Public Water Supply, Sweden. Emerg Infect Dis. 2014; 20: 581–589. doi: 10.3201/eid2004.121415 24655474 10.3201/eid2004.121415 PMC3966397

[13] Bouzid M, Hunter PR, Chalmers RM, Tyler KM. *Cryptosporidium* pathogenicity and virulence. Clin Microbiol Rev. 2013; 26: 115–34. doi: 10.1128/CMR.00076-12 23297262 10.1128/CMR.00076-12 PMC3553671

[14] IARC Working Group on the Evaluation of Carcinogenic Risks to Humans. Biological agents. Volume 100 B. A review of human carcinogens. IARC Monogr Eval Carcinog Risks Hum. 2012; 100: 1–441. PMC4781184 23189750

[15] Ruskoné-Fourmestraux A, Fischbach W, Aleman BM, Boot H, Du MQ, Megraud F et al EGILS consensus report. Gastric extranodal marginal zone B- cell lymphoma of MALT. Gut. 2011; 60: 747–758. doi: 10.1136/gut.2010.224949 21317175 10.1136/gut.2010.224949

[16] Perrone S D’Elia G M, Annechini G, Pulsoni A. Infectious Aetiology of Marginal Zone Lymphoma and Role of Anti-Infective Therapy. Mediterr J Hematol Infect Dis. 2016; 8: e2016006 doi: 10.4084/MJHID.2016.006 26740867 10.4084/MJHID.2016.006 PMC4696464

[17] Benamrouz S, Conseil V, Creusy C, Calderon E, Dei-Cas E, Certad G. Parasites and malignancies, a review, with emphasis on digestive cancer induced by *Cryptosporidium parvum* (Alveolata: Apicomplexa). Parasite. 2012; 19: 101–115. doi: 10.1051/parasite/2012192101 22348213 10.1051/parasite/2012192101 PMC3671432

[18] Certad G, Ngouanesavanh T, Guyot K, Gantois N, Chassat T, Mouray A et al *Cryptosporidium parvum*, a potential cause of colic adenocarcinoma. Infect Agent Cancer. 2007; 2: 22 doi: 10.1186/1750-9378-2-22 18031572 10.1186/1750-9378-2-22 PMC2217515

[19] Certad G, Creusy C, Ngouanesavanh T, Guyot K, Gantois N, Mouray A, et al Development of *Cryptosporidium parvum* induced gastro-intestinal neoplasia in SCID mice: severity of lesions is correlated with infection intensity. Am J Trop Med Hyg. 2010; 82: 257–265. doi: 10.4269/ajtmh.2010.09-0309 20134002 10.4269/ajtmh.2010.09-0309 PMC2813167

[20] Certad G, Creusy C, Guyot K, Mouray A, Chassat T, Delaire B, et al Fulminant cryptosporidiosis associated with digestive adenocarcinoma in SCID mice infected with *Cryptosporidium parvum* TUM1 strain. Int J Parasitol. 2010; 40: 1469–1475. doi: 10.1016/j.ijpara.2010.07.007 20708621 10.1016/j.ijpara.2010.07.007

[21] Benamrouz S, Conseil V, Chabé M, Praet M, Audebert C, Blervaque R, et al *Cryptosporidium parvum*-induced ileo-caecal adenocarcinoma and Wnt signaling in a mouse model. Dis Model Mech. 2014; 7: 693–700. doi: 10.1242/dmm.013292 24652769 10.1242/dmm.013292 PMC4036476

[22] Patel P, Hanson DL, Sullivan PS, Novak RM, Moorman AC, Tong TC, et al Adult and Adolescent Spectrum of Disease Project and HIV Outpatient Study Investigators. Incidence of types of cancer among HIV-infected persons compared with the general population in the United States, 1992–2003. Ann Intern Med. 2008; 148: 728–736. 18490686 10.7326/0003-4819-148-10-200805200-00005

[23] Shebl FM, Engels EA, Goedert JJ. Opportunistic intestinal infections and risk of colorectal cancer among people with AIDS. AIDS Res Hum Retroviruses. 2012; 28: 994–99. doi: 10.1089/AID.2011.0185 22149090 10.1089/aid.2011.0185 PMC3423655

[24] Sreedharan A, Jayshree RS, Sridhar H. Cryptosporidiosis among cancer patients: an observation. J Diarrhoeal Dis Res. 1996; 14: 211–213. 9019017

[25] Hassan SI, Sabry H, Amer NM, Shalaby MA, Mohamed NA, Gaballah H. Incidence of cryptosporidiosis in immunodeficient cancer patients in Egypt. J Egypt Soc Parasitol. 2002; 32: 33–46. 12049267

[26] K S R, Kumar K L R.: Intestinal Cryptosporidiosis and the Profile of the CD4 Counts in a Cohort of HIV Infected Patients. J Clin Diagn Res. 2013; 7: 1016–20. doi: 10.7860/JCDR/2013/5339.3062 23905093 10.7860/JCDR/2013/5339.3062 PMC3708188

[27] Izadi M, Jonaidi-Jafari N, Saburi A, Eyni H, Rezaiemanesh MR, Ranjbar R.: Prevalence, molecular characteristics and risk factors for cryptosporidiosis among Iranian immunocompromised patients. Microbiol Immunol. 2012; 56: 836–42. doi: 10.1111/j.1348-0421.2012.00513.x 23088420 10.1111/j.1348-0421.2012.00513.x

[28] Kirkpatrick BD, Noel F, Rouzier PD, et al: Childhood cryptosporidiosis is associated with a persistent systemic inflammatory response. Clin Infect Dis 2006; 43:604 doi: 10.1086/506565 16886154 10.1086/506565

[29] Rehg JE. Effect of interferon-gamma in experimental *Cryptosporidium parvum* infection. J Infect Dis. 1996; 174: 229 8656002 10.1093/infdis/174.1.229

[30] Tilley M, McDonald V, Bancroft GJ. Resolution of cryptosporidial infection in mice correlates with parasite-specific lymphocyte proliferation associated with both Th1 and Th2 cytokine secretion. Parasite Immunol. 1995; 17: 459 8552414 10.1111/j.1365-3024.1995.tb00915.x

[31] Sulżyc-Bielicka V, Kuźna-Grygiel W, Kołodziejczyk L, Bielicki D, Kładny J, Stepień-Korzonek M, Telatyńska-Smieszek B. Cryptosporidiosis in patients with colorectal cancer. J Parasitol. 2007; 93: 722–724. doi: 10.1645/GE-1025R1.1 17626376 10.1645/GE-1025R1.1

[32] Sulżyc-Bielicka V, Kołodziejczyk L, Jaczewska S, Bielicki D, Kładny J, Safranow K. Prevalence of *Cryptosporidium* sp. in patients with colorectal cancer. Pol Przegl Chir. 2012; 84: 348–351. doi: 10.2478/v10035-012-0058-4 22935456 10.2478/v10035-012-0058-4

[33] Baqai R, Anwar S, Kazmi SU. Detection of *Cryptosporidium* in immunosuppressed patients. J Ayub Med Coll Abbottabad. 2005; 17: 38–40. 16320794

[34] Dehkordy AB, Rafiei A, Alavi S, Latifi S. Prevalence of *Cryptosporidium* infection in immunocompromised patients, in South-West of Iran, 2009–10. Iran J Parasitol. 2010; 5: 42–47. PMC3279856 22347265

[35] Al-Qobati SA, Al-Maktari MT, Al-Zoa AM, Derhim M. Intestinal parasitosis among Yemeni patients with cancer, Sana'a, Yemen. J Egypt Soc Parasitol. 2012; 42: 727–34. 23469646 10.12816/0006356

[36] Sanad MM, Thagfan F, Al Olayan EM, Almogren A, Al Hammaad A, Al-Mawash A, Mohamed AA. Opportunistic Coccidian Parasites among Saudi Cancer Patients Presenting with Diarrhea: Prevalence and Immune Status. Res J Parasitol. 2014; 9: 55–63.

[37] Benamrouz S, Guyot K, Gazzola S, Mouray A, Chassat T, Delaire B, et al *Cryptosporidium parvum* Infection in SCID Mice Infected with Only One Oocyst: qPCR Assessment of Parasite Replication in Tissues and Development of Digestive Cancer. PLoS One. 2012; 7: e51232 doi: 10.1371/journal.pone.0051232 23272093 10.1371/journal.pone.0051232 PMC3521773

[38] Certad G, Benamrouz S, Guyot K, Mouray A, Chassat T, Flament N, et al Fulminant Cryptosporidiosis after Near-Drowning: a Human *Cryptosporidium parvum* Strain Implicated in Invasive Gastrointestinal Adenocarcinoma and Cholangiocarcinoma in an Experimental Model. Appl Environ Microbiol. 2012; 78: 1746–1751. doi: 10.1128/AEM.06457-11 22247151 10.1128/AEM.06457-11 PMC3298136

[39] Abdou AG, Harba NM, Afifi AF, Elnaidany NF. Assessment of *Cryptosporidium parvum* infection in immunocompetent and immunocompromised mice and its role in triggering intestinal dysplasia. Int J Infect Dis. 2013; 17: e593–600. doi: 10.1016/j.ijid.2012.11.023 23291034 10.1016/j.ijid.2012.11.023

[40] Heussler VT, Küenzi P, Rottenberg S. Inhibition of apoptosis by intracellular protozoan parasites. Int J Parasitol. 2001; 31: 1166–1176. 11563357 10.1016/s0020-7519(01)00271-5

[41] Carmen JC, Sinai AP. Suicide prevention: disruption of apoptotic pathways by protozoan parasites. Mol. Microbiol. 2007; 64: 904–916. doi: 10.1111/j.1365-2958.2007.05714.x 17501916 10.1111/j.1365-2958.2007.05714.x

[42] Kim SE, Paik HY, Yoon H, Lee JE, Kim N, Sung MK.: Sex- and gender-specific disparities in colorectal cancer risk.World J Gastroenterol. 2015; 21:5167–75. doi: 10.3748/wjg.v21.i17.5167 25954090 10.3748/wjg.v21.i17.5167 PMC4419057

[43] Sharma A, Greenman J, Walker LG, Monson JR.: Differences in cytokine levels due to gender in colorectal cancer patients. Cytokine. 2010; 50:91–3. doi: 10.1016/j.cyto.2010.01.002 20116278 10.1016/j.cyto.2010.01.002

[44] Pellegrini P, Contasta I, Del Beato T, Ciccone F, Berghella AM.: Gender-specific cytokine pathways, targets, and biomarkers for the switch from health to adenoma and colorectal cancer. Clin Dev Immunol. 2011; 2011: 819724 doi: 10.1155/2011/819724 22235223 10.1155/2011/819724 PMC3253453

[45] Espinoza JL, Minami M.: Sensing Bacterial-Induced DNA Damaging Effects via Natural Killer Group 2 Member D Immune Receptor: From Dysbiosis to Autoimmunity and Carcinogenesis. Front Immunol. 2018; 9:52 doi: 10.3389/fimmu.2018.00052 29422899 10.3389/fimmu.2018.00052 PMC5788971

